# Psychosocial and quality of life assessment in kidney transplant recipients: a focus on anxiety, depression, and clinical correlates

**DOI:** 10.1186/s12882-025-04418-3

**Published:** 2025-08-29

**Authors:** Mohammed Kamal Nassar, Eman Nagy, Mohamed Mohamed Elshial, Mostafa Mahmoud Samy, Mohamed Ali Eltamaly, Moustafa Nagi Elfarahati, Samar Tharwat

**Affiliations:** 1 Mansoura Nephrology and Dialysis Unit (MNDU), Department of Internal Medicine, Faculty of Medicine, Mansoura University, Mansoura, Egypt; 2 Faculty of Medicine, Mansoura University, Mansoura, Egypt; 3 Mansoura Manchester Medical Program, Faculty of Medicine, Mansoura University, Mansoura, Egypt; 4 Gastroenterology Unit, Department of Internal Medicine, Mansoura International Hospital, Egyptian Ministry of Health and Population, Mansoura, Egypt; 5 Rheumatology & Immunology Unit, Department of Internal Medicine, Faculty of Medicine, Mansoura University Hospital, El Gomhouria St, Mansoura, Dakahlia Governorate 35511 Egypt; 6 Department of Internal Medicine, Faculty of Medicine, Horus University, New Damietta, Egypt

**Keywords:** Anxiety, Depression, Kidney transplantation, Quality of life, Psychosocial factors

## Abstract

**Background:**

Kidney transplantation (KT) significantly improves survival and quality of life compared to dialysis, yet psychological disorders like anxiety and depression critically influence post-transplant outcomes.

**Objectives:**

To evaluate the prevalence, clinical correlates, and health-related quality of life (HRQoL) impacts of anxiety and depression in Kidney transplant recipients.

**Methods:**

A cross-sectional study of 161 adult Kidney transplant recipients at a tertiary care center assessed psychosocial and clinical parameters using the Hospital Anxiety and Depression Scale (HADS) and Kidney Disease Quality of Life − 36 (KDQOL-36). Sociodemographic, clinical, and laboratory data were analyzed with multivariate regression identifying predictors.

**Results:**

Abnormal anxiety and depression were observed in 24.3% and 9.9% of kidney transplant recipients, respectively. Anxiety correlated with elevated serum creatinine (*r* = 0.164, *p* = 0.039) and lower hemoglobin (*r* = -0.16, *p* = 0.043), while depression linked to lower hemoglobin (*r* = -0.172, *p* = 0.029) and longer transplant duration (*r* = 0.159, *p* = 0.045). Female gender, physical inactivity, psychiatric history, and higher creatinine independently predicted anxiety (all *p* < 0.05). Patients with abnormal anxiety or depression reported significantly poorer HRQoL, particularly in “burden of kidney disease” (65.17 ± 28.69) and mental health domains (44.85 ± 11.41).

**Conclusion:**

Anxiety and depression are prevalent in Kidney transplant recipients and strongly associated with clinical markers and impaired HRQoL. Routine psychosocial screening and targeted interventions, addressing anemia, physical activity, and mental health—are essential to optimize post-transplant care. Integrating biopsychosocial models into clinical practice may enhance adherence, graft survival, and quality of life in this vulnerable population.

**Clinical trial number:**

Not Applicable.

## Introduction

Chronic kidney disease (CKD) is a significant global health issue, affecting millions of people worldwide [1]. It is characterized by a gradual loss of kidney function, leading to complications such as cardiovascular diseases, anemia, and mineral bone disorders. The prevalence of CKD has been increasing globally, driven by factors such as population growth, aging, and the rising incidence of diabetes and hypertension [2]. According to the Global Burden of Disease Study 2021, the global prevalence of CKD was estimated to be 359 million cases in 2021, representing a 92% increase since 1990 [3]. CKD is a progressive condition that can ultimately lead to end-stage kidney disease (ESKD), at which point the kidneys are no longer able to function adequately on their own and renal replacement therapy—such as dialysis or kidney transplantation (KT)—is required for survival [4].

KT plays a crucial role in managing ESKD, offering significant advantages over dialysis in terms of survival, quality of life, and cost-effectiveness. It is recognized as the preferred treatment option, particularly for patients with chronic renal failure, as it can substantially reduce mortality rates associated with chronic dialysis programs [5]. The significance of psychosocial factors, particularly anxiety and depression, in post-transplant care is increasingly recognized as critical for improving patient outcomes [6]. These factors can adversely affect the quality of life, treatment adherence, and overall survival rates of kidney transplant recipients) [7].

The evidence overwhelmingly supports the superiority of KT over dialysis in terms of survival, quality of life, and cost-effectiveness [8]. Transplantation offers a substantial survival advantage that extends across all age groups, with benefits increasing over time after an initial perioperative risk period [9]. HRQoL improvements are comprehensive, affecting physical function, dietary freedom, independence, and reproductive health [10].

However, psychological disorders, particularly anxiety and depression, exert profound influences on post-transplant outcomes across adherence to treatment [11], health related quality of life (HRQoL), and graft survival. These factors interact with physiological processes, healthcare behaviors, and psychosocial dynamics, creating complex challenges for Kidney transplant recipients [8].

Despite advancements in understanding the clinical outcomes of Kidney transplant recipients, significant gaps remain in the psychosocial assessment of this population. Although the associations between anxiety, depression, and HRQoL in Kidney transplant recipients have been widely studied internationally, this work is among the first to provide a comprehensive, standardized analysis in an Egyptian cohort using validated instruments. Our findings fill a critical gap in local data, contextualize the psychosocial benefits of transplantation within Egypt, and identify modifiable predictors—such as physical inactivity and anemia—that can inform targeted interventions. This study aims to address these gaps by evaluating the correlates of anxiety and depression, assessing variations in HRQoL across psychosocial subgroups, and identifying predictors of poor mental health outcomes in Kidney transplant recipients.

## Patients and methods

### Study design and setting

This cross-sectional observational study was conducted at a tertiary care center and included 161 adult kidney transplant recipients aged ≥ 18 years who underwent KT between January 2023 and December 2024. Participants were recruited during routine outpatient follow-up visits to ensure representation of stable kidney transplant recipients at least six months post-transplant. Exclusion criteria encompassed multi-organ transplantation, active infections, or graft failure within six months post-surgery and those with incomplete or missing medical records. We also excluded any patient with pre-existing psychiatric disorders prior to KT.

### Sample size calculation

The sample size was calculated using the PASS program based on a previous study by Ashelleh et al. [12], which found a significant negative correlation (rho = -0.46, *p* ≤ 0.001) between anxiety and quality of life in patients with CKD. A sample size of 120 was initially estimated to provide a two-sided 95% confidence interval with a width of 0.299 for the Spearman’s correlation coefficient. However, to enhance statistical power, account for potential participant dropout or missing data, and allow for more precise subgroup analyses, we enrolled 161 patients. This larger sample size ensures greater reliability in detecting the association while maintaining robustness against variability in responses.

### Ethical consideration

The study protocol received ethical approval from the Institutional Research Board of the Faculty of Medicine at Mansoura University (approval registration number: R.24.06.2675), ensuring compliance with the ethical principles outlined in the Declaration of Helsinki. Written informed consent was obtained from all participants prior to enrollment, with detailed explanations of the study’s purpose, procedures, risks, and benefits. Confidentiality of participant data was rigorously maintained through anonymization of personal identifiers and secure storage of electronic records.

### Data collection

Data collection was conducted through structured face-to-face interviews and comprehensive medical record reviews. A trained researcher administered validated questionnaires, including the Hospital Anxiety and Depression Scale (HADS) and Kidney Disease Quality of Life Short Form (KDQOL-36), to all 161 participants during routine follow-up visits. Sociodemographic, clinical, and laboratory data—such as immunosuppressive regimens, serum creatinine, hemoglobin levels, and comorbidities—were systematically retrieved from electronic health records and written medical archives. This dual approach ensured the integration of patient-reported psychosocial outcomes with objective clinical parameters, enhancing data robustness. Interviews were standardized to minimize bias, while medical record extraction followed protocols to verify accuracy.

### Sociodemographic, clinical, therapeutic and laboratory data

Sociodemographic, clinical, therapeutic, and laboratory data were comprehensively collected for all kidney transplant recipients included in this study. Sociodemographic information such as age, gender, marital status, educational level, and area of residence was obtained during structured interviews with each participant. Clinical data encompassed comorbidities (including hypertension, diabetes, ischemic heart disease, and chronic obstructive pulmonary disease), transplant duration, and history of pre-transplant hemodialysis. Psychiatric comorbidity refers exclusively to psychiatric disorders that developed after transplantation (i.e., new-onset post-transplant psychiatric diagnoses), as identified through structured follow-up interviews and medical record review. Therapeutic details, including immunosuppressive regimens (prednisolone, tacrolimus, cyclosporine) and adjunctive medications, were also recorded. Laboratory parameters—such as serum creatinine, hemoglobin, intact parathyroid hormone (iPTH), and transferrin saturation (TSAT)—were retrieved from both electronic and written medical records.

### Hospital anxiety and depression scale

The HADS is a validated [13], self-report instrument designed to assess symptoms of anxiety and depression in medically ill populations, including kidney transplant recipients. It consists of 14 items, with 7 items each for anxiety and depression subscales, and each item is scored on a 4-point Likert scale ranging from 0 to 3, yielding subscale scores between 0 and 21. Higher scores indicate greater symptom severity, with commonly used cut-offs of 0–7 (normal), 8–10 (borderline), and 11–21 (abnormal) for each subscale.

### Kidney disease quality of life − 36

The KDQOL-36 was utilized to evaluate HRQoL in kidney transplant recipients [14]. This validated instrument included 36 questions about different aspects of quality of life capturing both generic and disease-targeted aspects of well-being. The tool assesses 5 domains: symptom/problem list, effects of kidney disease, burden of kidney disease, physical and mental health. “Symptom/problem list” refers to the KDQOL-36 domain assessing the degree of bother from 12 common kidney disease-related symptoms and problems (e.g., fatigue, pain, sleep disturbance, itching) experienced by patients during the past four weeks. Each domain is scored 0–100, with higher scores indicating better HRQoL. Composite scores for physical and mental health were derived using validated algorithms. The KDQOL-36 is particularly suited for transplant populations, as it addresses unique challenges such as treatment burden and immunosuppression side effects.

### Statistical analysis

Data were analyzed using SPSS v22.0, with continuous variables expressed as mean ± standard deviation (SD) or median (interquartile range, IQR) and categorical variables as frequencies (percentages). Group comparisons employed chi-square/Fisher’s exact tests for categorical data and ANOVA/Kruskal-Wallis tests for continuous variables, depending on data distribution. Correlations between HADS scores (anxiety/depression) and clinical/laboratory parameters were assessed using Pearson/Spearman coefficients. Univariate linear regression identified potential predictors of anxiety and depression, with covariates advanced to multivariate models. Significance was set at *p* < 0.05.

## Results

Table 1 presents the sociodemographic, clinical, therapeutic, and laboratory characteristics of the studied kidney transplant recipients (*n* = 161). The mean age of the cohort was 38.68 ± 13.93 years, with a majority being male (65.2%) and married (67.7%). Most patients resided in urban areas (76.4%) and had secondary school education (47.2%). Hypertension was the most common comorbidity (59%), followed by diabetes (23.6%). Immunosuppressive therapy predominantly included prednisolone (80%) and calcineurin inhibitors (90.7%), with tacrolimus being the most frequently used. Laboratory findings revealed a median serum creatinine level of 1.2 mg/dl and a mean blood hemoglobin level of 13.3 gm/dl.

**Table 1:** Sociodemographic, clinical, therapeutic and laboratory data of the studied patients (*n* = 161)

Variable	All
	(*n* = 161)
*Sociodemographic data*	
Age (years)	38.68 ± 13.93
*Gender*	
Male	105 (65.2)
Female	56 (34.8)
Married	109 (67.7)
Live alone	20 (12.4)
*Residence*:	
Urban	38 (23.6)
Rural	123 (76.4)
*Educational level*	
Illiterate	5 (3.1)
Primary school	7 (4.3)
Secondary school	76 (47.2)
College degree	72 (44.7)
Post-graduate	1 (0.6)
*Occupational status*	
Not working	44 (27.3)
Sick leave	4 (2.5)
Retired	28 (17.4)
Has a job	85 (52.8)
Physical activity:	
Active	86 (53.4)
Inactive	75 (46.6)
*Socioeconomic status*	
Low	65 (40.4)
Average	95 (59)
High	1 (0.6)
*Smoking*	
Non-smoker	121 (75.2)
Ex-smoker	35 (21.7)
Current smoker	5 (3.1)
Original kidney disease:	
DKD	6 (3.7)
Hypertensive nephropathy	37 (23)
Congenital (including ADPKD)	14 (8.7)
Glomerulonephritis	27 (16.8)
Drug-induced	13 (8.1)
Unknown	43 (26.7)
Others	21 (13)
Hemodialysis before kidney transplantation	128 (79.5)
Duration of hemodialysis (months)	12 (4–30)
Duration of kidney transplantation (months)	78.5 (44-119.5)
*Associated comorbidities*	
Diabetes	38 (23.6)
Hypertension	95 (59)
Psychiatric	4 (2.5)
COPD	3 (1.9)
IHD	8 (5)
*Immunosuppressive data*:	
Prednisolone	132 (80)
Dose (mg/day)	6.27 ± 3.19
Calcinurin inhibitors:	
Cyclosporine	15 (9.3)
Dose (mg/day)	125 (75–200)
Tacrolimus	146 (90.7)
Dose (mg/day)	2.75 (2-5.25)
Antiproliferative agents:	
Mycophenolic acid derivatives:	146 (90.7)
Mycophenolate mofetil Dose (mg/day)	1500 (1000–2000)1080 (720–1440)
Mycophenolate sodium Dose (mg/day)	10 (6.2)
Azathioprine:	100 (50–150)
Dose (mg/day)	5 (3.1)
Everolimus	1.5 (0.75–2.25)
Dose (mg/day)	
*Laboratory data*	
Serum creatinine (mg/dl)	1.2 (1.02–1.5)
Blood hemoglobin (gm/dl)	13.3 ± 1.93
Serum albumin (gm/dl)	3.96 ± 0.61
Serum calcium (mg/dl)	9 (8.5–9.3)
Serum phosphorus (mg/dl)	4 (3.6-5)
Tacrolimus trough level (pg/ml)	5.63 (4.42–7.22)
Uric acid (mg/dl)	6.2 (5.1–6.97)

HD (hemodialysis), COPD (chronic obstructive pulmonary disease), IHD (ischemic heart disease), iPTH (intact parathyroid hormone), TSAT (transferrin saturation)

The data represented as median (Q1-Q3), mean ± SD or number (percentage)

The HADS and HRQoL scores for the studied kidney transplant recipients are presented in Table 2. The median HADS-Anxiety score was 5 (Q1-Q3: 2–10), with 62.7% of patients classified as normal, 13% as borderline, and 24.3% as abnormal. For HADS-Depression, the median score was 3 (Q1-Q3: 1–7), with 75.2% of patients in the normal range, 14.9% borderline, and 9.9% abnormal. Regarding HRQOL domains, the highest scores were observed for the “Effect of kidney disease” (mean ± SD: 88.35 ± 15.87) and “Symptom/problem list” (84.77 ± 12.95), while the lowest scores were noted for the “Burden of kidney disease” (65.17 ± 28.69) and the composite scores for physical (46.42 ± 10.37) and mental health (44.85 ± 11.41).

**Table 2:** HADS score and KRQOL in the studied patients

Variable	The patients
	(*n* = 161)
*HADS score*	
HADS-Anxiety: median (Q1-Q3)	5 (2–10)
Normal	101 (62.7)
Borderline	21 (13)
Abnormal	39 (24.3)
HADS-Depression: median (Q1-Q3)	3 (1–7)
Normal	121 (75.2)
Borderline	24 (14.9)
Abnormal	16 (9.9)
*KRQOL domains*	
Symptom/problem list	84.77 ± 12.95
Effect of kidney disease	88.35 ± 15.87
Burden of kidney disease	65.17 ± 28.69
Physical health composite	46.42 ± 10.37
Mental health composite	44.85 ± 11.41

HADS (hospital anxiety and depression scale), KRQOL (kidney-related quality of life), MHD (maintenance hemodialysis)

The data presented as number (percentage) or median (Q1-Q3)

Table 3 presents a comparison of kidney transplant recipients across anxiety subgroups based on HADS-Anxiety scores. Female gender and a physically active lifestyle were significantly more prevalent among patients with higher anxiety levels (*p* < 0.001 and *p* = 0.019, respectively). Psychiatric comorbidities were notably more frequent in the abnormal anxiety group (*p* = 0.002). Additionally, serum creatinine levels were significantly higher in patients with borderline anxiety (*p* = 0.048). Other sociodemographic, clinical, and immunosuppressive therapy variables did not show significant differences between the anxiety subgroups.

**Table 3:** Comparison between anxiety subgroups regarding socio-demographic, medical and laboratory data

	HADS-Anxiety			
	Normal	Borderline	Abnormal	p
	(*n* = 101)	(*n* = 21)	(*n* = 39)	
***Sociodemographic data***				
Age	39.54 ± 14	34.14 ± 13.41	38.92 ± 13.91	0.234
*Gender*				**< 0.001**
Male	80 (79.2)	9 (42.9)	26 (66.7)	
Female	21 (20.8)	12 (57.1)	13 (33.3)	
Married	70 (69.3)	14 (66.7)	25 (64.1)	0.835
Live alone	14 (13.9)	2 (9.5)	4 (10.3)	0.77
Residence:				0.52
Urban	26 (25.7)	3 (14.3)	9 (23.1)	
Rural	75 (74.3)	18 (85.7)	30 (76.9)	
*Education*				0.195
Illiterate	5 (5)	0	0	
Primary school	3 (3)	2 (9.5)	2 (5.1)	
Secondary school	41 (40.6)	10 (47.6)	25 (64.1)	
College degree	51 (50.5)	9 (42.9)	12 (30.8)	
Post-graduate	1 (1)	0	0	
*Occupational status*				0.11
Not working	22 (21.8)	5 (23.8)	17 (43.6)	
Sick leave	3 (3)	0	1 (2.6)	
Retired	22 (21.8)	4 (19)	2 (5.1)	
Has a job	54 (53.5)	12 (57.1)	19 (48.7)	
Active lifestyle	46 (45.5)	12 (57.1)	28 (71.8)	**0.019**
*Socioeconomic status*				0.849
Low	43 (42.6)			
Average	57 (56.4)	7 (33.3)	15 (38.5)	
High	1 (1)	14 (66.7)	24 (61.5)	
		0	0	
*Smoking*				0.288
Non-smoker	72 (71.3)	16 (76.2)	33 (84.6)	
Ex-smoker	26 (25.7)	5 (23.8)	4 (10.3)	
Current smoker	3 (3)	0	2 (5.1)	
Hemodialysis before kidney transplantation	79 (78.2)	18 (85.7)	31 (79.5)	0.78
Duration of hemodialysis (months)				
	12 (4–36)	8 (4.5–18)	12 (3–30)	0.596
Duration of kidney transplantation (months)	74 (40.5–119)	95.5 (36.7–119)	84 (64–123)	0.457
***Comorbidities***				
Diabetes	23 (22.8)	7 (33.3)	8 (20.5)	0.51
Hypertension	57 (56.4)	15 (71.4)	23 (59)	0.44
Psychiatric	0	0	4 (10.3)	**0.002**
COPD	1 (1)	1 (4.9)	1 (2.6)	0.475
IHD	6 (5.9)	1 (4.8)	1 (2.6)	0.711
***Immunosuppressive data***				
Prednisolone	82 (81.2)	18 (85.7)	32 (82.1)	0.886
Dose (mg/day)	6.25 ± 3.55	5 (5–10)	5 (5-6.25)	0.607
Calcinurin inhibitors:				
Cyclosporine	11 (10.9)	1 (4.8)	3(7.7)125 (75–135)	**0.035**
Dose (mg/day)	150 (75–275)	-	36 (92.3)	0.317
Tacrolimus	90 (89.1)	20 (95.2)	3 (1-4.5)	0.627
Dose (mg/day)	2 (2–6)	5 (2-7.25)		0.388
Antiproliferative agents:				
Mycophenolic acid derivatives:	95 (94.1)	18 (85.7)	33 (84.6)	0.159
Mycophenolate Mofetyl	1500(1250–2000)	1250 (1000–1500)		0.392
Dose (mg/day)			1250 (1000–1875)	
Mycophenolate sodium	1080 (720–1440)	1080 (1260–1440)		0.132
Dose (mg/day)	4	3	540 (360–1080)	0.185
Azathioprine:	50 (75–100)	100 (50–100_	3	0.882
Dose			100 (50–100)	
***Laboratory data***				
Serum creatinine (mg/dl)	1.3 ± 0.58	1.49 ± 0.77	1.3 (1.1–1.9)	**0.048**
Blood hemoglobin (gm/dl)	13.58 ± 1.68	12.83 ± 2.15	12.83 ± 2.32	0.295
Serum albumin (gm/dl)	4.3 ± 0.35	3.76 ± 0.25	3.5 ± 0.98	0.211
Serum calcium (mg/dl)	9 (8-9.22)	9 (8.6–9.2)	9 (8.65–9.4)	0.565
Serum phosphorus (mg/dl)	4 (3.6-5)	4.1 (3.5-5)	4 (3.6–4.46)	0.53
Tacrolimus trough level (pg/ml)	5.6 (4.3–7.18)	5.8 (4.4–9.1)	5.6 (4.7–7.1)	0.672
Uric acid (mg/dl)	6.05 (5.1–6.92)	5.9 (4.2–6.25)	6.8 (5.5–8.3)	0.227

HD (hemodialysis), COPD (chronic obstructive pulmonary disease), IHD (ischemic heart disease), iPTH (intact parathyroid hormone), TSAT (transferrin saturation)

The data represented as median (Q1-Q3), mean ± SD or number (percentage)

Table 4 compares sociodemographic, clinical, immunosuppressive, and laboratory parameters among kidney transplant recipients stratified by HADS-depression subgroups. No significant differences were observed in age, gender, residence, marital status, lifestyle, or most comorbidities across the groups. However, patients with abnormal depression scores showed a trend toward higher serum creatinine levels (*p* = 0.057) and lower hemoglobin levels (*p* = 0.093). Tacrolimus use was common across all groups, while cyclosporine was used only in the normal and borderline groups (*p* = 0.016). Overall, the table highlights limited but notable clinical variations among kidney transplant recipients with differing levels of depressive symptoms.

**Table 4:** Comparison between depression subgroups regarding socio-demographic, medical and laboratory data

	HADS-Depression			
	Normal	Borderline	Abnormal	p
	(*n* = 121)	(*n* = 24)	(*n* = 16)	
***Sociodemographic data***				
Age	38.71 ± 13.63	35.75 ± 16.24	42.81 ± 12.13	0.255
*Gender*				0.254
Male	83 (68.7)	14 (58.3)	8 (50)	
Female	38 (31.4)	10 (41.7)	8 (50)	
Married	79 (65.3)	17 (70.8)	13 (81.3)	0.412
Live alone	15 (12.4)	3 (12.5)	2 (12.5)	1
Residence:				0.763
Urban	27 (22.3)	7 (29.2)	4 (25)	
Rural	94 (77.7)	17 (70.8)	12 (75)	
*Education*				0.582
Illiterate	5 (4.1)	0	0	
Primary school	6 (5)	1 (4.2)	0	
Secondary school	51 (42.1)	15 (62.5)	10 (62.5)	
College degree	58 (47.9)	8 (33.3)	6 (37.5)	
Post-graduate	1 (0.8)	0	0	
*Occupational status*				0.807
Not working	34 (28.1)	4 (16.7)	6 (37.5)	
Sick leave	3 (2.5)	1 (4.2)	0	
Retired	21 (17.4)	4 (16.7)	3 (18.8)	
Has a job	63 (52.1)	15 (62.5)	7 (43.8)	
Active lifestyle	66 (54.5)	12 (50)	8 (50)	0.883
*Socioeconomic status*				0.146
Low	49 (40.5)	11 (45.8)	5 (31.3)	
Average	72 (59.5)	12 (50)	11 (68.8)	
High	0	1 (4.2)	0	
*Smoking*				0.739
Non-smoker	89 (73.6)	20 (83.3)	12 (75)	
Ex-smoker	28 (23.1)	3 (12.5)	4 (25)	
Current smoker	4 (3.3)	1 (4.2)	0	
Hemodialysis before kidney transplantation	95 (78.5)	20 (83.3)	13 (81.3)	0.889
Duration of hemodialysis (months)				
	12 (3–30)	9 (6–24)	24 (4.5–51)	0.73
Duration of kidney transplantation (months)	74 (42.5-112.5)	92.5 (38.5-141.75)	89.5 (64.75–123)	0.367
***Comorbidities***				
Diabetes	27 (22.3)	8 (33.3)	3 (18.8)	0.454
Hypertension	66 (54.5)	16 (66.7)	13 (81.3)	0.088
Psychiatric	2 (1.7)	1 (4.2)	1 (6.3)	0.458
COPD	2 (1.7)	1 (4.2)	0	0.598
IHD	5 (4.1)	3 (12.5)	0	0.142
***Immunosuppressive data***				
Prednisolone	97 (80.2)	21 (87.5)	14 (87.5)	0.578
Dose (mg/day)	6.03 ± 3.26	7.5 ± 3.53	7.5 ± 2.88	0.242
Calcinurin inhibitors:				
Cyclosporine	13 10.7	1 (4.2)	1 (6.2)	**0.016**
Dose	150 (72.5–225)	NA	NA	0.317
Tacrolimus	108 (89.3)	23 (95.8)	15 (93.8)	0.542
Dose	2 (2–5)	5 (2-6.5)	5 (2.5-8)	0.158
Antiproliferative agents:				
Mycophenolic acid derivatives:	111 (91.7)	21 (87.5)	14 (87.5)	0.727
Mycophenolate Mofetyl: Dose (mg/day) :	1500 (1000–2000)	1250 (750–1500)	1500 (1000–1500)	0.4
Mycophenolate sodium:				
Dose (mg/day)	1080 (720–1440)	1080 (720–1440)	1440 (720–1440)	0.226
Azathioprine:	8	1	1	0.902
Dose	100 (50–100)	50	100	0.374
***Laboratory data***				
Serum creatinine (mg/dl)	1.2 (1-1.49)	1.18 (1.1–1.74)	1.46 (1.12–2.33)	0.057
Blood hemoglobin (gm/dl)	13.54 ± 1.72	12.57 ± 2.15	12.65 ± 2.7	0.093
Serum albumin (gm/dl)	4 ± 0.66	4.3 ± 0.42	3.5 ± 0.42	0.63
Serum calcium (mg/dl)	9 (8.1–9.4)	9 (8.9–9.3)	9 (7.37–9.17)	0.791
Serum phosphorus (mg/dl)	4 (3.6-5)	4 (3.7–4.5)	4 (3.6-5)	0.87
Tacrolimus trough level (pg/ml)	5.6 (4.4–7.07)	6.3 (4.5–8.62)	6.05 (4.82–8.06)	0.638
Uric acid (mg/dl)	5.9 (5.1–6.82)	6 (4–9)	6.5 (5.25–7.95)	0.634

Table 5 presents the correlations between HADS-anxiety and depression scores and various clinical and demographic variables. A weak but statistically significant positive correlation was observed between HADS-anxiety scores and serum creatinine levels (*r* = 0.164, *p* = 0.039), while a negative correlation was found with blood hemoglobin levels (*r* = -0.16, *p* = 0.043). For HADS-depression scores, a significant positive correlation was noted with the duration of KT (*r* = 0.159, *p* = 0.045), and a negative correlation with blood hemoglobin levels (*r* = -0.172, *p* = 0.029).

**Table 5:** Correlations of HADS-anxiety and depression scores

	HADS Anxiety		HADS Depression	
	Correlation coefficient (*r*)	*P*	Correlation coefficient (*r*)	*p*
Age (year)	-0.062	0.434	0.041	0.609
Duration of HD before kidney Tx. (month)	0.117	0.141	-0.003	0.975
Duration of kidney Tx. (month)	0.045	0.617	0.159	**0.045**
Serum creatinine (mg/dl)	0.164	**0.039**	0.149	0.062
Blood hemoglobin (gm/dl)	-0.16	**0.043**	-0.172	**0.029**
Serum albumin (gm/dl)	-0.538	0.071	-0.385	0.216
Trough level of FK (pg/ml)	0.05	0.531	0.071	0.37
Uric acid (mg/dl)	-0.067	0.695	-0.071	0.678

Table 6 presents the results of univariate and multivariate linear regression analyses assessing factors associated with anxiety levels among kidney transplant recipients. In the multivariate analysis, female gender (B = -3.63, *p* < 0.001), active lifestyle (B = 1.626, *p* = **0.028**), presence of psychiatric disorders (B = 8.251, *p* < 0.001), and elevated serum creatinine levels (B = 0.791, *p* = 0.012) emerged as significant predictors of increased anxiety scores. Although cyclosporine use and hemoglobin level were significant in univariate analysis, they did not retain significance in the multivariate model.

**Table 6:** Univariate and multivariate linear regression analysis of HADS-anxiety and HADS-depression

	HADS-anxiety							HADS-depression				
	Univariate analysis		Multivariate analysis					Univariate analysis				
Variable	B	P value	Beta	B	P value	Confidence interval for B		Beta	B	P value	Confidence interval for B	
						Lower bound	Higher bound				Lower bound	Higher bound
Gender:												
Female	R		R	R	R							
Male	-4.576	**< 0.001**	-0.339	-3.63	**< 0.001**	-5.28	-1.97					
Active lifestyle	1.829	**0.024**	0.158	1.626	**0.028**	0.175	3.077					
Psychiatric disorders	10.385	**< 0.001**	0.253	8.251	**< 0.001**	3.7	12.79					
Cyclosporine use	-2.634	**0.05**	-0.02	-0.34	0.784	-2.78	2.1	-0.001	-0.009	0.993	-2.148	2.129
Seum creatinine	0.978	**0.004**	0.184	0.791	**0.012**	0.176	1.406					
Blood hemoglobin	-0.488	**0.019**	-0.007	-0.018	0.933	-0.426	0.391	-0.199	-0.423	**0.011**	-0.749	-0.096
Duration of kidney transplantation									0.004	0.254	-0.003	0.011

Table 6 presents the univariate linear regression analysis identifying variables associated with HADS-depression scores among kidney transplant recipients. A statistically significant inverse relationship was found between blood hemoglobin levels and depression scores (B = -0.423, *p* = 0.011), indicating that lower hemoglobin levels were associated with higher depressive symptoms. In contrast, neither the duration of KT (B = 0.004, *p* = 0.254) nor the use of cyclosporine (B = -0.009, *p* = 0.993) showed a significant association with depression scores.

Table 7 demonstrates significant differences in HRQoL domains among kidney transplant recipients based on their anxiety levels. Patients with abnormal HADS-anxiety scores reported the poorest outcomes across all HRQOL domains, including symptom/problem list, burden, and both physical and mental health composites (all *p* < 0.05). In contrast, those with normal anxiety scores exhibited significantly better HRQOL, highlighting the negative impact of anxiety on post-transplant well-being.

**Table 7:** Health-related quality of life data of studied patients across HADS-anxiety and depressionsubgroups

	HADS-anxiety subgroups				HADS-depression subgroups			
	Normal	Borderline	Abnormal	P	Normal	Borderline	Abnormal	P
	(*n* = 101)	(*n* = 21)	(*n* = 39)		(*n* = 121)	(*n* = 24)	(*n* = 16)	
Symptom/problem list	91.67	77.08	79.17	**< 0.001**	89.58	75	75	**< 0.001**
	(83.33–97.92)	(62.5–87.5)	(72.39–85.42)		(81.25–95.83)	(58.33–84.89)	(58.33–85.42)	
Effect of kidney disease	96.88	87.5	90.63	**0.001**	93.75	81.25	85.94	**< 0.001**
	(87.5–100)	(60.94–93.75)	(78.13–96.88)		(87.5–100)	(71.88–96.88)	(57.03–96.09)	
Burden of kidney disease	81.25	50	43.75	**< 0.001**	75	50	43.75	**< 0.001**
	(56.25–93.75)	(34.37-75)	(25–75)		(50-93.75)	(31.25–79.68)	(6.25–59.37)	
Physical health composite	50.74	38.29	44.32	**0.029**	52.47	35.47	40.11	**< 0.001**
	(42.43–55.67)	(33.58–54.68)	(35-54.64)		(43.02–56.3)	(28.5-39.08)	(34.98-47)	
Mental health composite	50.3	40.34	35.31	**< 0.001**	48.54	36.08	32.66	**< 0.001**
	(44.88–56.83)	(27.47–45.7)	(28.1-44.81)		(43.36–55.45)	(28.35–42.17)	(22.48–45.21)	

The data represented as median (Q1-Q3)

As shown in Table 7, kidney transplant recipients with higher levels of depressive symptoms exhibited significantly lower HRQoL scores across all assessed domains. Patients in the abnormal depression subgroup reported the lowest median scores in the symptom/problem list, effect and burden of kidney disease, as well as in both physical and mental health composite scores (*p* < 0.001 for all), indicating a clear negative association between depressive symptoms and perceived quality of life.

## Discussion

This study demonstrates a substantial psychosocial burden among kidney transplant recipients, with 24.3% exhibiting abnormal anxiety and 9.9% showing abnormal depression according to HADS criteria. Notably, higher anxiety scores were significantly associated with elevated serum creatinine and lower hemoglobin levels, while depressive symptoms correlated with both lower hemoglobin and longer transplant duration. Female gender, physical inactivity, and the presence of psychiatric comorbidities were associated with increased anxiety scores. Importantly, both anxiety and depression were associated with impairments in HRQoL, with the most pronounced deficits observed in the “burden of kidney disease” and mental health domains. Patients with abnormal anxiety or depression reported the lowest scores across nearly all HRQoL domains, underscoring the critical interplay between psychosocial distress and perceived well-being in this population. These findings highlight the need for routine psychological assessment and targeted interventions to improve both mental health and overall quality of life in kidney transplant recipients.

The relatively young age of our cohort and the high proportion of hypertensive ESKD are consistent with national data from Egypt, where earlier onset of ESKD and predominance of hypertension as a cause have been documented [15, 16]. All transplants in this study were from living donors, reflecting current Egyptian practice, as deceased donor transplantation remains rare due to persistent cultural and regulatory challenges [17].

The observed prevalence of anxiety in our cohort of kidney transplant recipients was 24.3% for abnormal anxiety, with an additional 13% classified as borderline, indicating that more than one-third of patients experience at least moderate anxiety symptoms post-transplant. This rate is within the wide range reported in the literature, where studies have documented anxiety prevalence between 12.5% and 50% among kidney transplant recipients, depending on assessment tools and population characteristics [18, 19]. For example, Arapaslan et al. reported anxiety in 50% of renal transplant recipients, while other cohorts have found lower rates, such as 12.5% and 11.3% in separate studies using the HADS [18]. An Iranian study using the Beck Anxiety Inventory found that 50% of kidney transplant recipients had anxiety, with 27% experiencing mild, 15.5% moderate, and 7.5% severe symptoms [19]. A Brazilian study reported that depression and anxiety symptoms were more prevalent among dialysis patients (41.7% and 32.3%, respectively) than among transplant recipients (13.3% and 20.3%, respectively), using the Beck Inventory [20]. Karaminia and colleagues found that anxiety scores were significantly lower in transplant recipients compared to HD patients, while depression scores did not differ significantly between the two groups [21].

This study identified a 9.9% prevalence of abnormal depression among kidney transplant recipients, with 14.9% classified as borderline. These rates align with HADS-based studies from Panama (11.8%) and Thailand (12.9%) [22], reflecting the tool’s specificity in excluding somatic symptoms that often inflate depression scores in medically ill populations. However, they contrast sharply with higher rates reported in studies using instruments like the Beck Depression Inventory (BDI), such as 41.4% in Japan [23] and 22.2% in China [24], where somatic complaints (e.g., fatigue, insomnia) are included in assessments. For instance, an Iranian study using BDI reported 75% depression prevalence in kidney transplant recipients [19], underscoring how methodological differences drive variability.

The lower prevalence in this Egyptian cohort compared to some Middle Eastern studies may also reflect cultural factors, such as stigma around mental health reporting or stronger familial support structures. Additionally, the cohort’s younger mean age (38.7 years) and higher education levels (47.2% secondary school) may buffer against severe depression, contrasting with older, less-educated populations in other studies [19].

This study identified female gender as a significant predictor of higher anxiety scores (B = -3.63, *p* < 0.001). This aligns with broader literature indicating that women are more susceptible to anxiety disorders due to biological, hormonal, and sociocultural factors [25]. In the context of KT, women may face additional stressors such as caregiving responsibilities, body image concerns related to immunosuppressive therapy (e.g., weight gain, hirsutism), and gender-specific disparities in healthcare access [26]. These findings underscore the need for gender-sensitive mental health screening and interventions tailored to address the unique psychosocial challenges faced by female transplant recipients.

The study identified a significant positive correlation between elevated serum creatinine levels and anxiety in kidney transplant recipients (*r* = 0.164, *p* = 0.039), with multivariate analysis confirming serum creatinine as an independent predictor of anxiety (β = 0.791, *p* = 0.012). This association likely operates through bidirectional pathways. Physiologically, subclinical graft dysfunction—indicated by rising creatinine—may trigger sympathetic nervous system activation, elevating cortisol and norepinephrine levels, which exacerbate anxiety and perpetuate a stress-inflammatory cycle detrimental to graft health [27]. Psychologically, patients often interpret creatinine increases as precursors to graft failure, fostering hypervigilance and catastrophic thinking [28]. This aligns with findings by Hu et al. (2021), who noted that anxiety impairs self-management behaviors critical for creatinine clearance, though their study emphasized depression’s stronger correlation with renal function [18].

This study identified a significant inverse correlation between hemoglobin levels and depressive symptoms in kidney transplant recipients (*r* = -0.172, *p* = 0.029), with lower hemoglobin independently predicting higher HADS-depression scores in regression models. This aligns with prior research showing an association between anemia and depressive symptoms, potentially mediated by factors such as fatigue, reduced physical activity, and neurocognitive changes [29]. Notably, even mild hemoglobin reductions (within normal ranges) were associated with worse mental health outcomes, suggesting a dose-dependent relationship [30]. Mechanistically, iron deficiency—common in kidney transplant recipients due to post-transplant blood loss, inflammation, and immunosuppressive drug effects—may impair dopamine and serotonin synthesis, critical neurotransmitters regulating mood [31]. Recent studies highlight iron deficiency (independent of anemia) as a driver of depressive symptoms in transplant populations, with iron’s role in mitochondrial function and oxygen delivery to limbic brain regions further linking hematologic parameters to psychological well-being [30, 32]. For instance, Kremer et al. (2024) found iron deficiency doubled the risk of major depressive symptoms in kidney transplant recipients, even after adjusting for hemoglobin levels [33].

The study identified a weak but statistically significant positive correlation between transplant duration and depression scores (HADS-D: *r* = 0.159, *p* = 0.045), suggesting that longer time post-transplant may exacerbate depressive symptoms. However, this association did not retain significance in multivariate regression analysis (*p* = 0.254), indicating that transplant duration itself may not directly drive depression but could interact with cumulative psychosocial or clinical stressors. For instance, prolonged exposure to immunosuppressant side effects (e.g., cosmetic changes, financial strain) and diminishing social support over time may erode coping resilience [34]. This aligns with Tsunoda et al. (2010), who found no direct link between transplant duration and depression in Japanese kidney transplant recipients, emphasizing instead the role of comorbid conditions and marital discord [23]. Conversely, Hu et al. (2021) reported that longer transplant duration correlated with poorer self-management, indirectly fueling depressive symptoms through non-adherence. The discrepancy between correlation and regression findings in this study may reflect confounding by unmeasured variables, such as evolving graft function or socioeconomic shifts [18]. Clinically, these results highlight the need for sustained mental health monitoring even in long-term kidney transplant recipients, as late-onset depression could signal unaddressed psychosocial burdens or subclinical graft dysfunction.

Although the correlations between anxiety, depression, and clinical parameters (e.g., serum creatinine, hemoglobin) reached statistical significance, the observed coefficients (*r* ≈ 0.16–0.17) indicate only a weak association. This suggests that, while these relationships are unlikely to be due to chance, their practical or clinical impact is limited. The findings should therefore be interpreted as hypothesis-generating rather than as evidence of robust predictive value. Further research, ideally with longitudinal designs and larger effect sizes, is needed to clarify the clinical relevance of these associations [35].

The findings of this study underscore the imperative for routine psychosocial screening integrated into standard post-transplant care protocols. Implementing validated tools like the HADS and KDQOL-36 during follow-up visits enables early identification of anxiety/depression, particularly in high-risk subgroups such as women, physically active patients, and those with psychiatric histories. Targeted interventions should include: gender-specific mental health programs addressing hormonal and caregiving stressors in female kidney transplant recipients; structured lifestyle interventions (e.g., supervised aerobic/resistance training) to mitigate anxiety linked to inactivity; and iron supplementation protocols for patients with hemoglobin < 13.5 g/dl to alleviate depressive symptoms mediated by cerebral hypoxia [36]. Multidisciplinary teams should prioritize preemptive psychiatric referrals for borderline cases (14.9% depression, 13% anxiety) to prevent progression to clinical disorders. Additionally, patient education initiatives demystifying creatinine fluctuations and digital health tools (e.g., creatinine-tracking apps with clinician feedback) could reduce hypervigilance-driven anxiety. These strategies, informed by the study’s predictors and HRQoL deficits, align with evidence showing psychosocial support improves adherence by 45% and graft survival by 25% in structured programs [37, 38]. A paradigm shift toward integrated biopsychosocial care models is essential to optimize long-term outcomes in kidney transplant recipients.

This study’s strengths include its cross-sectional design with standardized, validated tools (HADS, KDQOL-36), ensuring reliable psychosocial assessment tailored to transplant populations. The integration of clinical (e.g., serum creatinine, hemoglobin), therapeutic (immunosuppressive regimens), and psychosocial data provides a multidimensional analysis of post-transplant outcomes, addressing a critical gap in holistic care research.

However, limitations must be acknowledged. The generalizability of our findings is constrained by several factors. First, the study population was relatively young, predominantly male, and largely urban-based, which may not represent the full spectrum of kidney transplant recipients in other regions or healthcare systems. Second, the single-center design further limits external validity, as center-specific practices, resources, and patient demographics can influence both clinical and psychosocial outcomes. Third, socioeconomic and cultural factors—such as income, education, healthcare access, and community support—can significantly impact both mental health and transplant outcomes. Another major limitation of this study is the absence of a control group, such as patients on dialysis or healthy controls. This restricts our ability to determine whether the observed prevalence of anxiety, depression, and impaired HRQoL is specific to the post-transplant population or reflects general trends among patients with chronic illnesses. Future research should incorporate appropriate control groups to enable direct comparisons and more robust causal inferences regarding the psychosocial impact of kidney transplantation.

While efforts were made to minimize selection bias by enrolling consecutive patients during routine follow-ups, the exclusion of unstable kidney transplant recipients (e.g., recent graft failure) may underrepresent severe mental health burdens. The cross-sectional design precludes causal inference, as temporal relationships between variables (e.g., hemoglobin-depression links) remain speculative. Longitudinal studies are needed to delineate whether anxiety/depression drive graft dysfunction or vice versa. Additionally, unmeasured confounders—such as detailed socioeconomic stressors or medication side-effect profiles—may influence observed associations. Despite these constraints, the study provides actionable insights for integrating psychosocial screening into transplant care, while underscoring the need for multicenter, prospective cohorts to validate these findings.

Future research should prioritize longitudinal cohort studies to delineate causal relationships between psychosocial disorders (anxiety/depression) and clinical outcomes in kidney transplant recipients. Prospective designs tracking HADS scores, HRQoL domains, and laboratory markers (e.g., creatinine, hemoglobin) over 5–10 years could clarify whether mental health declines precede graft dysfunction or vice versa. Additionally, randomized controlled trials (RCTs) are needed to evaluate targeted interventions, such as cognitive-behavioral therapy (CBT) protocols tailored to kidney transplant recipients’ unique stressors (e.g., fear of rejection, immunosuppressant side effects) and iron supplementation regimens for patients with subclinical deficiency.

In conclusion, this study highlights the substantial impact of psychosocial disorders, particularly anxiety and depression, on the well-being of kidney transplant recipients. Nearly one-third of patients experienced clinically significant anxiety or depression, with these conditions strongly associated with poorer health-related quality of life, higher serum creatinine, and lower hemoglobin levels. Female gender, physical inactivity, psychiatric history, and elevated serum creatinine emerged as significant predictors of psychological distress. Importantly, both anxiety and depression were associated with marked impairments across all domains of HRQoL, underscoring the intricate interplay between mental health and clinical outcomes in this population. These findings emphasize the necessity of routine psychosocial screening and targeted interventions as integral components of post-transplant care. Addressing mental health alongside traditional biomedical management is essential to optimize adherence, enhance quality of life, and ultimately improve long-term outcomes for kidney transplant recipients.

## Data Availability

The datasets used and/or analysed during the current study are available from the corresponding author on reasonable request.

## Abbreviations

ADPKD: Autosomal Dominant Polycystic Kidney Disease
BDI: Beck Depression Inventory
BDNF: Brain-Derived Neurotrophic Factor
CBT: Cognitive-Behavioral Therapy
CKD: Chronic Kidney Disease
COPD: Chronic Obstructive Pulmonary Disease
DKD: Diabetic Kidney Disease
ESKD: End-Stage Kidney Disease
HADS: Hospital Anxiety and Depression Scale
HRQoL: Health-Related Quality of Life
HPA: Hypothalamic-Pituitary-Adrenal
IHD: Ischemic Heart Disease
iPTH: Intact Parathyroid Hormone
KDQOL-36: Kidney Disease Quality of Life-36
KT: Kidney Transplantation
RCT: Randomized Controlled Trial
SD: Standard Deviation
SF-36: 36-Item Short Form Health Survey
TSAT: Transferrin Saturation
